# Predictive value of systemic inflammatory index (SII) for the time to negative nucleic acid conversion in patients with mild COVID-19 by the omicron wave

**DOI:** 10.3389/fmed.2024.1474236

**Published:** 2025-05-01

**Authors:** Yuyan Fan, Ning Yang, Jialu Zhuo, Ting Han

**Affiliations:** ^1^Department of Clinical Nutrition, Shanghai Public Health Clinical Center, Fudan University, Shanghai, China; ^2^Department of Clinical Nutrition, Shanghai Tenth People’s Hospital, Tongji University of Medicine, Shanghai, China; ^3^Shanghai Clinical Nutrition Quality Control Center, Shanghai Tenth People’s Hospital, Tongji University of Medicine, Shanghai, China

**Keywords:** COVID-19, omicron variant, nucleic acid negative time, systemic inflammatory index, AUC

## Abstract

**Objective:**

Inflammatory indices are pivotal markers in gaging the harm of the COVID-19 trajectory. The definitive impact of inflammatory indices on forecasting the period required for a negative shift in nucleic acid status during the Omicron wave remains ambiguous. This research endeavors to delineate the relationship between the Systemic Inflammatory Index (SII) and the timeline for conversion to negative nucleic acid status in Omicron variant-infected patients, and to scrutinize the prognostic validity of SII for such conversion.

**Methods:**

Adult patients hospitalized at the Shanghai Public Health Clinical Center with mild cases attributed to the Omicron variant were studied from March to December 2022. They were stratified into early-conversion (with mild cases attributed to (>10 days) groups). Analyzing patient information, clinical traits, and laboratory results, we divided patients into two groups. We used logistic regression to find the link between SII and virus test timing and built ROC curves to measure predictive value via AUC.

**Results:**

A total of 2,603 patients were enrolled. Univariate analysis found big differences in pulse rates, respiratory rates, prealbumin levels, HS-CRP levels, IL-6 levels, SII, and PNI (*p* < 0.05) between the groups. Adjusting for confounders, logistic regression revealed that the highest SII group had a 1.46 greater risk of not clearing a 10-day PCR test than the lowest group (OR = 1.46; 95% CI, 1.173–1.817, *p* = 0.001). Each one-unit rise in SII raised the risk of 10-day PCR failure by 0.1% (*p* < 0.0001). The ROC curve showed SII’s AUC as 0.603 (95% CI: 0.576–0.630), predicting virus test turn-around with a cut-off of 920.5, 61.9% specificity, and 52.5% sensitivity. Compared to other indicators such as IL-6 and HS-CRP, SII exhibited the highest AUC value and specificity.

**Conclusion:**

In mild cases caused by the Omicron wave, there was a discernible link between the SII and the period leading to a negative nucleic acid test outcome, with higher SII values indicating an increased risk of prolonged conversion time. SII might help guide treatment better than other indicators by predicting disease course.

## Introduction

The severe acute respiratory syndrome coronavirus 2 (SARS-CoV-2) leads to novel coronavirus pneumonia 2019 (COVID-19), a severe respiratory disease with potentially fatal outcomes (1). The advent of the Omicron strain has been marked by its pronounced contagiousness, ledding to the rapid global predominance of this variant over Delta (2, 3). Consequently, Omicron quickly supplanted Delta as the most prevalent form of the virus internationally. By the end of February 2022, Shanghai in China grappled with a substantial influx of cases driven by the Omicron variant. Studies have revealed that a significant number of patients exhibit a clustering of their viral genetic material in the SARS-CoV-2 Omicron surge (4).

Reverse transcription-polymerase chain reaction (RT-PCR) is crucial for determining discharge and self-isolation protocols. Predicting the time to negative conversion is crucial for determining optimal retesting intervals to prevent unnecessary healthcare costs and prolonged isolation periods due to repetitive testing. The Omicron variant spreads rapidly, with a higher proportion of mild cases and shorter average hospital stay durations (3, 5). The time to nucleic acid conversion is also critical for monitoring disease progression and optimizing treatment options; studies have shown that severe patients have a longer time to nucleic acid conversion (6). Despite severe patients typically exhibiting delayed conversion times, there is a lack of knowledge concerning potential predictors for this conversion in Omicron variant-infected individuals. Hence, an urgent necessity for dependable, cost-effective, and swift biomarkers to forecast the time to negative nucleic acid conversion.

Most COVID-19 patients experience immune dysregulation (7). However, litthle is known about the relationship between clinical patterns, systemic non-specific markers of inflammation, and the immune response. Previous reported modification in severe forms of COVID-19 showed increased levels of C-reactive protein, interleukin-6, and D-dimers to predict mortality in hospitalized COVID-19 patients (8, 9). At the onset of COVID-19, many patients were found to have abnormalities in specific blood cell counts, such as increased neutrophils, decreased lymphocytes, and decreased platelets (10). The Systemic Immune-Inflammatory Index (SII) is a biomarker that consolidates counts of platelets, lymphocytes and neutrophils, readily accessible through standard blood analyses. While previous research has linked SII to predicting COVID-19 severity and mortality (11, 12), its potential as a prognostic factor for predicting the time to nucleic acid conversion is still ambiguous. Policy decisions for preventing COVID-19 infection are still mainly guided by strategies based on reducing the time to negative viral conversion.

The objective was to investigate the correlation between the SII and mild COVID-19 infections caused by the Omicron variant, as well as to evaluate its predictive efficacy.

## Methods

### Study design and population

The population consisted of 2,603 adult inpatients with the Omicron variant of COVID-19 between March and December 2022. Inclusion: (1) mild infection diagnosis following the 10th version of the National COVID-19 protocol, with primary respiratory symptoms and absent specific radiological signs of COVID-19 pneumonia; (2) being at least 18 years old; (3) having complete medical documentation, inclusive of nucleic acid testing while hospitalized; (4) obtaining informed consent and voluntary engagement. Exclusions: (1) patients with a diagnosis of moderate to severe infection per the National COVID-19 protocol’s 10th revision; (2) Age < 18 or pregnant; (3) cases with missing clinical or medical record information at the time of admission; (4) individuals with multiple hospital readmissions. The study protocol was approved by the Research Ethics Committee of the Shanghai Public Health Clinical Center (No. 2022-S118-02).

### Data consolidation

All data were obtained from patient electronic records in the hospital’s HIS system. Data collection included comprehensive general information (name, gender, age, marital history, comorbidities, etc.), biochemical indicators (lymphocyte count, platelet count, albumin, pre-albumin, creatinine, glucose, etc.), inflammatory markers (HS-CRP, IL-6), and others (BMI, pulse, breathing rate, etc.). Blood samples for laboratory tests were collected with cephalic venipuncture and then examined in the hospital laboratory using standard clinical chemical analysis. Calculation of the SII was derived from the parameters of a complete blood count where SII = platelet count × neutrophil count / lymphocyte count. The prognostic nutritional index (PNI) was calculated based on biochemical indicators, where PNI = serum albumin +5 × lymphocyte count.

### Study outcomes

All patients enrolled underwent immediate nucleic acid testing upon admission, with the initial positive result establishing the time of positive nucleic acid detection. Defining Viral Shedding Time (VST) involves calculating the time from hospital entry to the attainment of two back-to-back negative nucleic acid tests, separated by a minimum of 24 h, to confirm the readiness for deisolation (13, 14). The average viral shedding time among all patients was 10.4 ± 3.8 days. Based on that, patients were divided into two groups: 1318 individuals (50.6%) in the early viral conversion group (≤10 days) and 1,285 individuals (49.4%) in the late viral conversion group (>10 days). The duration of the conversion process varied, with the shortest period being as little as 3 days, while the longest spanned up to 31 days.

### Statistical methods

Analyses in our study were handled by Statistical Package for the Social Sciences 25.0 (SPSS 25.0, IBM, USA). Descriptive statistics were utilized to characterize the study subjects. We show continuous variables with normal distribution as the mean ± standard deviation. ANOVA compared normal continuous variables between groups; the Wilcoxon rank-sum test was for others. We used counts and percentages for categorical variables and the chi-square test to compare groups. Multivariable logistic regression models were utilized for the multivariate analysis to examine the relationship between SII and the speed of nucleic acid conversion in three distinct models. Model 1 had no adjustments. Model 2 adjusted (age, gender, comorbidities and BMI). Model 3, built on Model 2, additionally adjusted for pulse, respiratory rate, prealbumin, creatinine, glucose, and PNI. The receiver operating characteristic (ROC) curve checked SII, IL-6, and HS-CRP as predictors, with the Youden index picking the cutoff for sensitivity and specificity. Statistical significance was established for *p*-values that fell below 0.05.

## Results

### Clinical characteristics

A total of 2,603 patients with mild diagnosis according to the 10th edition of the national guidelines for COVID-19 management were included. Table 1 shows the Comprehensive general information on the enrollments. The average age of the study population was 43 ± 16 years, with 1,067 males (41.0%) and 1,536 females (59.0%). Compared with the early-conversion group, patients in the late-conversion group had higher HS-CRP levels, IL-6, and lower PNI and pre-albumin. In addition, SII was significantly higher in the late conversion group than in the early conversion group (583.71 ± 380.48 vs. 826.79 ± 712.25, *p* < 0.001).

**Table 1:** Comprehensive general information by virus shedding duration (>10 days).

	Early VST group (*n* = 1,318)	Late VST group (*n* = 1,285)	*p*-value
Age (years)	43.18 ± 16.36	43.51 ± 16.75	0.612
Gender, *N* (%)			0.445
Male	528 (20.3%)	496 (19.1%)	
Female	790 (30.3%)	789 (30.3%)	
Marital status, *N* (%)			0.979
Married	982 (37.7%)	958 (36.8%)	
Unmarried	336 (12.9%)	327 (12.6%)	
Hypertension, *N* (%)	112 (4.3%)	122 (4.7%)	0.361
Diabetes, *N* (%)	73 (2.8%)	89 (3.4%)	0.132
BMI (kg/m^2^)	23.71 ± 4.01	23.55 ± 3.87	0.305
Pulse (bpm)	86.94 ± 14.54	92.33 ± 14.53	<0.001
Respiration rate (bpm)	19.07 ± 5.71	19.70 ± 8.69	0.027
Albumin (g/L)	43.14 ± 3.23	43.22 ± 3.31	0.491
Pre-albumin (mg/L)	231.34 ± 61.48	210.23 ± 56.80	<0.001
Hemoglobin (g/L)	141.67 ± 16.96	142.37 ± 16.91	0.292
White blood cell count (× 10^9^ /L)	6.22 ± 1.96	5.63 ± 1.79	<0.001
Neutrophil count (× 10^9^ /L)	3.82 ± 1.65	3.72 ± 1.59	0.097
Lymphocyte count (× 10^9^ /L)	1.69 ± 0.67	1.16 ± 0.54	<0.001
Platelet count (× 10^9^ /L)	223.52 ± 62.27	198.41 ± 52.88	<0.001
Creatinine (μmol/L)	75.63 ± 29.63	77.49 ± 32.54	0.126
Glucose (mmol/L)	6.35 ± 2.34	6.47 ± 2.57	0.015
IL-6 (pg/mL)	2.01 ± 7.43	2.83 ± 5.75	0.002
HS CRP (mg/L)	8.11 ± 11.55	9.55 ± 11.88	0.014
PNI (%)	51.57 ± 4.98	49.03 ± 4.45	<0.001
SII	583.71 ± 380.48	826.79 ± 712.25	<0.001

### Association of SII with the time to virus turn-negative duration

The highest tertile SII was linked to a longer time for the virus to clear in COVID-19 patients, with statistical significance (OR = 1.46; *p* = 0.001) (Table 2). Higher levels of SII may be associated with failure to 10-day negative nucleic acid conversion. In addition, SII as a continuous variable, adjusted for confounders, showed that the risk of failure to 10-day negative nucleic acid conversion was elevated by 0.1% for each 1-unit increase (*p* < 0.001).

**Table 2:** Multivariate logistic regression analysis of the relationship between SII and viral shedding time > 10 days.

Variant	Crude model	Model 1	Model 2
Continuous variable			
	1.001 (1.001, 1.001)	1.001 (1.001, 1.001)	1.001 (1.000, 1.001)
	*p* < 0.001	*p* < 0.001	*p* < 0.001
Categorical variable			
Tertile 1	Reference	Reference	Reference
Tertile 2	1.181 (0.977, 1.427)	1.198 (0.990, 1.449)	1.110 (0.905, 1.362)
	*p* = 0.086	*p* = 0.063	*p* = 0.316
Tertile 3	2.003 (1.654, 2.425)	2.020 (1.666, 2.448)	1.460 (1.173, 1.817)
	*p* < 0.001	*p* < 0.001	*p* = 0.001
Trend *p*-value	*p* < 0.001	*p* < 0.001	0.002

Model 1: adjusted by age, sex, BMI and comorbidity.

Model 2: adjusted by model1 plus heart rate, breathing rate, pre-albumin, creatinine, glucose and PNI.

### ROC curves and efficacy of SII and other inflammatory markers for predicting 10-day negative nucleic acid conversion

**Figure 1:**
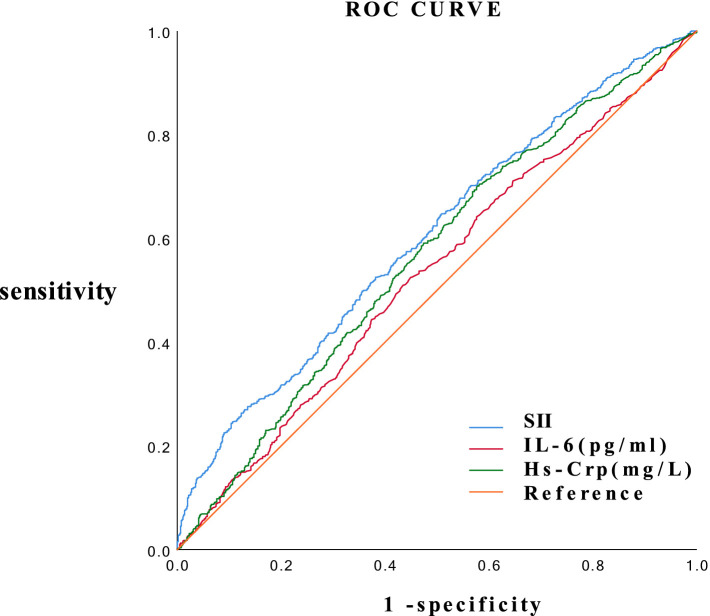
ROC curves for SII and other metrics to predict negative viral shedding time.

SII and other inflammation levels were evaluated using ROC curves (AUC) to predict virus exit time. The AUCs for SII, IL-6, and HS-CRP were 0.603 (95% CI: 0.576–0.630), 0.533 (0.505–0.561), and 0.567 (0.539–0.595), respectively (Figure 1). Compared to IL-6 and HS-CRP, the best cut-off value for SII to predict 10-day negative nucleic acid conversion was 920.5, with the highest specificity of 61.9%; IL-6 was the next highest at 47.6%; and HS-CRP was the lowest at 42.2% (Table 3).

**Table 3:** Diagnostic performance of inflammatory indices in determining COVID-19 nucleic acid negative conversion time.

	Specificity	Sensitivity	AUC	95% CI	*p*-value
SII	61.9%	52.5%	0.603	0.576–0.630	*p* < 0.001
IL-6	47.6%	52.4%	0.533	0.505–0.561	0.023
HS-CRP	42.2%	70.1%	0.567	0.539–0.595	*p* < 0.001

## Discussion

In our study, we investigated the correlation between the SII and the duration required for asymptomatic or mild COVID-19 patients to achieve a negative nucleic acid test during the Omicron surge. Our results indicate that an elevated SII is linked to a prolonged period until a negative test outcome is observed in these individuals. Moreover, the SII demonstrated superior predictive accuracy compared to other markers of inflammation.

The SII is an important inflammatory marker, providing an in-depth representation of the host’s immune and inflammatory condition. SII, as an index describing the instability of inflammatory responses, has been suggested as a follow-up prognostic indicator for many diseases such as tumors, chronic diseases, and severe pneumonia (15–18). During COVID-19, due to the increase in non-specific cellular responses, patients’ clinical conditions also dynamically change, which are similar to other aforementioned diseases. As longer nucleic acid-negative conversion time in COVID-19 patients affects poor prognosis, shortening the viral shedding time has been recognized as an effective assessment of patient prognosis (6). Thus, this study is the first one to explore whether SII determines nucleic acid-negative by 10 days.

Previous studies have revealed a correlation between a high SII value and the severity of COVID-19 (19). However, limited evidence exists regarding the relationship between SII and nucleic acid-negative time. A prior study demonstrated that patients with decreased platelet, leukocyte, and lymphocyte levels faced an elevated risk of delayed viral clearance (20). Elevated SII values, serving as a composite indicator involving specific hematological cells, signify an immune regulation and systemic inflammation imbalance. This imbalance is likely to compromise the immune system’s response to viral infections, potentially leading to prolonged viral clearance times. Consistent with this hypothesis, in this study, the SII of the late nucleic acid conversion group was higher than the early conversion group. Further analysis using multivariable logistic regression revealed that COVID-19 patients with the highest SII levels had a notably longer time to test negative compared to those with the lowest levels. For each unit increase in SII, there was a 1.45 times greater risk of the virus shedding lasting over 10 days. This underscores the key role of a person’s individual immune-inflammatory state in determining the length of time it takes for nucleic acid tests to convert from positive to negative. While this study has endeavored to address certain confounding variables, there may still be some overlooked factors that warrant further investigation.

Currently, most studies on SII and COVID-19 focus on predicting the severity of COVID-19 using SII values, with very few studies investigating the duration of negative nucleic acid clearance by detecting SII values. For example, in the study by Nalbant et al., ICU inpatients had significantly higher SII values, with a sensitivity of 70.8% and specificity of 66.0% in detecting the severity of COVID-19 (AUC = 0.689, 95% CI: 0.559–0.819, with ≥813.6 as the optimal cutoff point) (21). Xue et al. found that patients with severe conditions had significantly higher SII levels (1,263.52) compared to those with non-severe conditions (618.35). With a cutoff value of 809.02, the SII showed a sensitivity of 72.41% and a specificity of 67.86% (22). For instance, in a study by Nalbant et al., ICU patients with COVID-19 exhibited markedly higher Systemic Immune-Inflammation Index (SII) values. These values demonstrated a sensitivity of 70.8% and a specificity of 66.0% in discerning the severity of the disease, with an area under the receiver operating characteristic curve (AUC) of 0.689 (95% confidence interval: 0.559–0.819), and an optimal cutoff point of ≥813.6 for distinguishing severe cases. Xue et al. observed that SII levels were significantly elevated in patients with severe COVID-19 when compared to those with non-severe forms of the illness (1,263.52 vs. 618.35). The sensitivity and specificity of SII in identifying severe cases at the optimal cutoff value of 809.02 were 72.41 and 67.86%, respectively. These findings highlight the utility of SII as a potential biomarker for assessing COVID-19 severity. Considering the relationship between SII and the prolonged time to nucleic acid conversion in COVID-19, we investigated the predictive value of SII for the time of negative viral shedding. The results indicated that compared to the other two inflammatory markers, HS-CRP and IL-6, SII had higher predictive value (AUC = 0.603) with a higher sensitivity (52.5%) and specificity (61.9%) at the optimal cutoff value of 920.5, suggesting it could be a convenient and accessible predictive tool.

Clinical reports of COVID-19 patients show significant activation of T lymphocytes and monocyte-macrophages, leading to the expression of many cytokines, especially IL-6, further causing a cytokine storm and severe inflammatory reactions. This study found that the IL-6 levels were higher in the late nucleic acid conversion group. Many COVID-19 studies show that severe cases often have higher IL-6 levels, which can predict the disease’s severity (8). This is primarily due to the immune dysregulation or macrophage activation syndrome of SARS-CoV-2, both characterized by pro-inflammatory cytokines, where immune dysregulation is driven by IL-6. Global studies have found that IL-6 has the highest specificity in predicting severe COVID-19 patients and is associated with poor clinical outcomes (23). However, its role in predicting the time of nucleic acid-negative conversion remains unclear. In this study, the AUC for IL-6 in predicting the viral shedding time by 10 days among COVID-19 patients was 0.533, with both specificity and sensitivity lower than SII.

HS-CRP is a nonspecific inflammatory marker unaffected by radiation therapy, chemotherapy, and glucocorticoids. When the body experiences inflammatory damage, bacterial infections, etc., oxidative stress causes a rapid increase in HS-CRP levels, distinguishing between bacterial and viral infections. As a critical pro-inflammatory cytokine, HS-CRP triggers a cascade reaction and amplifies the cytokine storm, making it a potential, reliable, and easily applicable predictor of acute phase COVID-19 prognosis (24). A meta-analysis, encompassing a total of 16 studies and 3,962 COVID-19 patients, has revealed that individuals with non-severe cases of the virus exhibited significantly lower levels of high-sensitivity HS-CRP when contrasted with those in the severe group (25). This study also made a similar finding that the group with prolonged time to nucleic acid conversion had higher HS-CRP levels, suggesting that HS-CRP may be involved in the progression of COVID-19. When analyzing COVID-19 cancer patients, Zhou et al. found that CRP was an important risk factor for COVID-19 mortality and plotted a probability of death curve based on these two factors, with an AUC of 0.918 (26–30). In this study, when using HS-CRP levels as an inflammatory marker to predict the speed of nucleic acid conversion, the AUC was 0.567, indicating a high sensitivity but poor specificity compared to SII and IL-6.

A rapid clinical diagnosis is crucial in symptomatic treatment and patient isolation to prevent the transmission of COVID-19. Despite some widely recognized challenges, for example long testing time and no PCR laboratory equipment in community, the PCR test is still the gold standard. Other widely used techniques, such as biochemical and complete blood count analyses, may be faster, easy to measure, and low-cost techniques that aid in the diagnosis and prognosis of COVID-19. Efficient and rapid forecasting programs can be used as a complement to response measures to local outbreak risk surges. Their efficacy can play a role in triage, and their application is equally evident in the protection of emergency care resources in health care facilities. This study has some limitations, including being a single-center cohort study, having a retrospective design and lacking anthropometric data due to the urgency of epidemic disease. Hence, more extensive prospective studies are required.

In conclusion, SII is a valuable and cost-effective biomarker compared to other inflammatory markers, relating to how fast COVID-19 changes its genetic material and forecasting how the illness gets worse. When SII ≥920.5, the time to nucleic acid conversion may be prolonged, aiding clinicians in identifying patients at risk of slow nucleic acid conversion and poorer prognosis in the early stages of COVID-19. Our study demonstrated a correlation between the two; in the future, further studies combining inflammatory state measures with overall survival modeling may provide more clinical relevance to the diagnosis and prognosis of COVID-19.

## Data Availability

The original contributions presented in the study are included in the article/supplementary material, further inquiries can be directed to the corresponding authors.
